# Alligators in the big city: spatial ecology of American alligators (*Alligatormississippiensis*) at multiple scales across an urban landscape

**DOI:** 10.1038/s41598-020-73685-x

**Published:** 2020-10-06

**Authors:** Eli R. Beal, Adam E. Rosenblatt

**Affiliations:** University of North Florida, 1 UNF Dr, Jacksonville, FL 32224 USA

**Keywords:** Urban ecology, Behavioural ecology

## Abstract

Urbanization impacts wildlife, yet research has been limited to few taxa. American alligators (*Alligator* *mississippiensis*) are apex predators that have received minimal attention within urban areas. We investigated potential effects of urban land use on alligators through surveys of relative alligator abundance in nine tributaries of the lower St. Johns River within Jacksonville, FL. We then explored the potential effects of urban development on alligator spatial distribution and habitat selection at coarse and fine scales. At the coarse scale, we found no correlation between percent developed land and alligator abundance across tributaries; instead, salinity was the primary driver. However, at the fine scale alligators preferred habitats with more open water and vegetated shorelines and avoided anthropogenic structure. Surprisingly, only one of 93 sighted individuals was an adult. Hunting and nuisance alligator data suggests that adults are relatively rare in Jacksonville because they have been targeted for removal. Thus, smaller alligators still occupy urban habitats because they are not targeted and face no competition from adults. Increasing urbanization and human activity may further degrade alligator habitats and limit the distribution of breeding adults, potentially leading to local population declines.

## Introduction

A major driver of land use change is urbanization, whereby the land surface of relatively small areas is hyper-developed to support high-density human populations. This type of development is a force of biotic homogenization, where the environment built to meet the relatively narrow needs of humans creates more homogenous habitat and species assemblages^1^. Changes in habitat structure would therefore be expected to greatly influence the ecology of organisms in cities. Urban areas are one of the fastest growing types of land use, with the size of these areas expected to increase 139% in the southeast U.S. alone by 2060^2^. Despite the rapid growth of urban areas, our understanding of the ecological effects of urbanization is still in its infancy. Filling this knowledge gap will be key for moving toward the development and implementation of sustainable urban growth practices.

One group of organisms that has been largely overlooked in the field of urban ecology is large predators. They are typically excluded from areas of dense human habitation, especially in developing regions, due to the costs associated with their presence such as human and livestock endangerment^3^. If large predators can find a way to subsist in an urban environment, they are faced with many challenges. For example, the limited availability and fragmented nature of suitable habitat in urban areas has been shown to limit intraspecific variation in predator home range size^4^, possibly leading to the exclusion of individuals that require larger ranges. If suitable habitat can be found, urban predators can also face higher densities of conspecifics in these areas^5^. Despite the challenges associated with living alongside humans, some species of predators persist in urban areas, however these tend to be small- to medium-bodied mesopredators like raccoons (*Procyon* *lotor*), red foxes (*Vulpes* *vulpes*), and coyotes (*Canis* *latrans*), which display cryptic behaviors^5^. In contrast, large predators like leopards (*Panthera* *pardus*) and spotted hyenas (*Crocuta* *crocuta*) are more frequently documented in peri-urban and rural areas where they rely almost exclusively on domestic animals for food^6,7^. Changes in land use within peri-urban and rural areas has also been found to affect the level of human-wildlife conflict with large predators like black bears (*Ursus* *americanus*^8^). Despite the direct effects that large predators can have on humans and their domestic animals in peri-urban and rural areas, little research has been performed in highly urbanized areas.

The American alligator (*Alligator* *mississippiensis*) is a widely abundant, large-bodied apex predator found across the southeastern U.S. but has received minimal attention within urban areas. This is particularly surprising because alligators, and crocodilians in general, are regularly sighted within 10 km of city centers^9^. Furthermore, alligators are a well-known indicator species that have been used to track the health of other ecosystems like the Everglades due to their ability to integrate changes in habitat and water quality within their tissues and behaviors^10^. To our knowledge no studies have yet been published that investigate alligator spatial ecology in a heavily urban landscape, despite relatively large increases in the number of reported nuisance alligator complaints and alligator bites on humans over the last few decades^11^. However, there have been two studies in “urban-influenced” areas: Eversole et al.^12^ investigated habitat selection and distribution of an alligator population in a nature preserve on the outskirts of Houston, TX and found that alligators tended to avoid areas with the highest levels of human activity. Similarly, Lewis et al.^13^ investigated alligator habitat selection and distribution in a nature preserve on the outskirts of Fort Worth, TX and found that alligator behaviors may have been impacted by boat traffic.

Our study took place along the St. Johns River, an iconic part of the Florida landscape. The water system is a source of sustenance and employment across 12 counties, and the waters support abundant and diverse flora and fauna. The river also runs directly through Jacksonville, the largest city by land area in the contiguous U.S. and the largest city by population in Florida. Previous studies have shown that urban development around this river has shifted overall ecosystem function through the alteration of hydrology, chemistry, and biotic richness^14^. The health of the St. Johns River is also threatened by pollution, over-use, and mismanagement^15^. Monitoring programs for some species of animals and plants have been initiated in this region^15^, but alligators have received minimal attention from researchers within the lower St. Johns River system.

We hypothesized that alligators found in the lower St. Johns River system would avoid areas that have become intensively urbanized because of the associated alteration of natural habitat features and increased levels of human activity. We expected alligator density to instead be highest in the least developed areas, and in terms of habitat selection, we hypothesized that alligators would show avoidance of anthropogenic structure. Urban development alters the habitat that alligators have evolved in for millions of years, therefore we expected that any deviation in habitat quality, from an alligator’s perspective, would influence their spatial ecology patterns.

## Results

### Distribution

We recorded a total of 93 alligator sightings during nighttime spotlight surveys across time and space (Table 1). Size classification was heavily skewed towards juveniles and sub-adults with only one individual falling into the 180–270 cm size class. The remaining individuals with confirmed total length estimations fell into the 30–90 cm size class (n = 50), the 90–180 cm size class (n = 12), or were coarsely estimated as less than 180 cm (n = 6). The remaining 24 individuals submerged before we could estimate total length. We found alligators in all tributaries at least once during the year except in Clapboard Creek, the least urbanized water system that is also closest to the Atlantic Ocean. The summer season contained the most alligator sightings (n = 58). We encountered fewer animals in the spring season (n = 22), and even fewer in the fall and winter (n = 8 and n = 5, respectively).

**Table 1:** Number of alligator sightings by tributary (listed by tributary from northeast to south) over the span of a year, separated by season in which the sighting occurred.

Tributary	Winter sightings	Spring sightings	Summer sightings	Fall sightings	Average sightings
Clapboard	0	0	0	0	0.00
Dunn	0	0	0	1	0.25
Broward	0	0	3	0	0.75
Trout	0	1	0	0	0.25
Arlington	1	5	13	2	5.25
Ortega	0	4	14	0	4.50
Doctors	0	4	6	3	3.25
Julington	2	5	4	2	3.25
Black	2	3	18	0	5.75

When investigating relative alligator abundance, we did not find any of the candidate explanatory variables to always be statistically significant across seasons and tributaries. However, we found salinity to be significant in three of the four sampling seasons and in the global dataset as well (all P ≤ 0.035). We also found upland nonforested land was positively correlated with relative alligator abundance in three of the five datasets. We found other land use types and environmental conditions such as air temperature to significantly affect relative alligator abundance in a smaller number of datasets, but not consistently. Because we found no land use type to be a consistently significant factor at one buffer size and percent coverage of individual land use types were highly correlated across buffer sizes, we only report analyses of land use at the 1 km buffer size. We could only generate multiple linear regression models for the spring and summer seasons as well as the global dataset based on the normality of their distributions. Salinity once again appeared to be a major driving force, but other covariates such as the level of ambient light and the presence of forested and nonforested land use types also appeared as significant factors in the spring season models (Table 2). The ranking of models by their AIC_c_ values confirms that salinity is the most important predictor of relative alligator abundance across tributaries (Table 2).

**Table 2:** Significant multiple linear regression models for normally distributed alligator sightings that incorporate environmental cofactors and various measures of land use (LU) at levels of 0.1, 1, 3, and 5 km buffers surrounding each transect.

Data set	Model parameters	P	|AIC_c_|
**Spring sightings**			
Environmental data only	Salinity + light	0.001	0.532
	Salinity	0.004	6.094
0.1 km LU	Salinity	0.001	0.532
	Salinity + light	< 0.001	5.501
	Salinity + light + forests LU	0.004	6.094
1 km LU	Salinity + forests LU	0.001	0.805
	Salinity	0.004	6.094
3 km LU	Salinity + forests LU	0.001	0.557
	Salinity	0.004	6.094
5 km LU	Nonforested LU + salinity	0.002	1.843
	Nonforested LU + salinity + light	0.001	3.843
	Nonforested LU	0.003	5.762
**Summer sightings**			
All data sets	Salinity	0.035	–
**Average sightings**			
All data sets	Salinity	0.003	–

Specific data sets are outlined for spring sightings since model parameters were variable across buffer sizes. Summer and average sightings each generated one significant model with salinity as the only parameter across all LU buffer sizes. AIC_c_ values are used to rank spring sightings models within a data set where a smaller absolute value indicates a more parsimonious model.

To ensure that the effects of salinity were not biased by environmental outliers, we removed the two most tidally influenced and saltiest tributaries (Clapboard Creek and Dunn Creek) from the dataset and repeated both sets of analyses. Upon removing these two, the number of variables we found to be correlated with relative alligator abundances was greatly reduced. We still found salinity to be a statistically significant predictor in the spring season and in the averaged global dataset. Stepwise multiple linear regression analyses and subsequent AIC_c_ model ranking for the tidally unbiased data still found salinity and air temperature to be significant predictive variables (all P ≤ 0.017).

### Habitat selection

Surveys of used and available alligator habitats produced a total of 89 paired data points across time and space. We found statistically significant differences between the used and available habitat within the data analysis groups. Using all data across time and space, we found alligators inhabited areas with greater expanses of open water, minimal anthropogenic structure, and heavily vegetated shorelines (Table 3). Results from using all data collected within a season across space were subject to inter-season variation, but anthropogenic structure was almost always avoided by sighted alligators (Table 4). On average, alligators were found more than 50 m from the nearest anthropogenic structure.

**Table 3:** Results of Wilcoxon signed rank tests from the global dataset that independently compared all habitat characteristics from alligator used habitat to their respective value in available habitat.

Habitat characteristic	Used habitat		Available habitat		P
	x̄ (%)	SE	x̄ (%)	SE	
Open water	57	2.2	51	1.6	0.007*
Emergent vegetation	10	1.7	13	1.7	0.19
Floating vegetation	10	1.9	12	1.9	0.10
Anthropogenic structure	4	1.1	8	1.2	< 0.001*
Dry ground	19	2.0	16	1.7	0.56
Shoreline vegetation	87	3.2	81	3.5	0.007*

Average percent of each habitat type is represented for both used and available habitat. Statistically different results are marked with an asterisk (*).

**Table 4:** Results of Wilcoxon signed rank tests and paired samples t-tests independently comparing each habitat characteristic from the used habitat to the available habitat within a given season.

Season	Habitat characteristic	P	Average percent characteristic (used habitat)	Average percent characteristic (available habitat)
Winter (n = 5)	Open water	0.59	34	32
	Emergent vegetation	0.88	20	19
	Floating vegetation	1.00	29	29
	Anthropogenic structure	0.66	9	7
	Dry ground	0.14	8	14
	Shoreline vegetation	0.32	100	88
Spring (n = 22)	Open water	0.031*	63	52
	Emergent vegetation	0.31	10	12
	Floating vegetation	0.14	7	9
	Anthropogenic structure	0.017*	5	11
	Dry ground	0.48	16	17
	Shoreline vegetation	0.26	80	79
Summer (n = 54)	Open water	0.33	56	52
	Emergent vegetation	0.067	7	13
	Floating vegetation	0.37	11	12
	Anthropogenic structure	0.001*	3	7
	Dry ground	0.029*	22	16
	Shoreline vegetation	0.037*	89	82
Fall (n = 8)	Open water	0.23	61	53
	Emergent vegetation	0.22	22	14
	Floating vegetation	0.10	0	4
	Anthropogenic structure	0.34	9	14
	Dry ground	0.024*	8	15
	Shoreline vegetation	0.22	91	77

Statistically different results are marked with an asterisk (*).

## Discussion

The lower St. Johns River system has not escaped the ever-expanding influence of urbanization. Tributaries such as the Arlington River, for example, are surrounded by land of which only 13% is considered undisturbed (not used for urban, agriculture, or transportation purposes or left barren by human influence). Large predators in areas such as these are subject to intense anthropogenic pressures and have historically received little recognition or study, perhaps because they were assumed to be nonexistent. Our study demonstrates that one species of large predator, the American alligator, can still inhabit dense urban areas but that the spatial ecology and body size range of the species may be altered by shifts in land use and human activity.

At a coarse scale, alligator distribution within the lower St. Johns River system appears to be largely dependent on salinity, with alligators avoiding saltier tributaries across all seasons. Even more compelling, analyses which did not include the two most tidally influenced tributaries still found salinity to be the strongest predictor of relative alligator abundance. This result is not particularly surprising since it is consistent with our existing understanding of alligator sensitivity to salinity^16–22^.

While salinity appears to be the primary driver of alligator distribution, we also found air temperature to be a significant predictor of alligator abundance in several cases at a coarse scale. Again, this is expected because warmer air temperatures are known to positively influence the number of alligators in a given area, especially when incorporating seasonality into analyses^18,23,24^. Additionally, we did find that some land use types, such as forested and nonforested areas, were significant predictors of alligator abundance in certain situations, but were subject to high levels of multicollinearity and failed to consistently appear in multiple linear regression models across data sets. Land use patterns may therefore have some effect on alligator distribution at a coarse scale but to a far lesser degree than that of environmental factors like salinity or temperature.

At the finer scale of alligator habitat selection, our data suggests that individuals prefer more natural habitat features and tend to avoid anthropogenic structure within tributaries. Specifically, alligators tended to select areas with more open water and shoreline vegetation. These factors have been reported to be important for other alligator populations in settings with less human impacts^20,21,25,26^. When statistically significant differences were observed in the proportion of anthropogenic structure, there was always less structure in the used habitat than in available habitat. Although no previous study has been performed in a predominantly urban setting, alligator abundance, and crocodilian abundance more broadly, has been shown to be reduced in areas that are affected by human presence and activity, consistent with our results^12,27^. Neither emergent nor floating vegetation differed significantly between used and available habitats consistently, indicating little to no preference. However, the presence of emergent and floating vegetation is known to affect detectability in crocodilian spotlight surveys^13,28,29^. Tributaries we surveyed were bimodal in that they either had prevalent or minimal aquatic vegetation. Tributaries containing large amounts of aquatic vegetation, such as Black Creek, supported some of the largest alligator populations. If we underestimated alligator abundance in these areas because of limited detectability, corrections would only strengthen the results of this study.

We also found an incredibly strong bias toward sightings of small alligators across all tributaries. With 98.6% of all size-classified individuals falling below the length of 180 cm, adults were remarkably absent from the tributaries. This result is particularly surprising since a previous study in a human-disturbed area found no differences in habitat selection between alligator size classes and little segregation between size classes^12^. The most likely explanation for our result is that adult alligators in the lower St. Johns River system have been mostly removed by hunters or nuisance alligator trappers over time, and the small number of remaining adults has learned to strongly avoid urban areas and human activity. Hunter harvest data from the Florida Fish and Wildlife Conservation Commission (FWC; myfwc.com/wildlifehabitats/wildlife/alligator/harvest/) shows that between 2011 and 2018, 155 alligators were harvested in Duval County, which covers the same area as the city of Jacksonville. The yearly average total length of the harvested alligators in Duval County never exceeded 245 cm, while 83% of the other counties in Florida had at least one yearly average total length of harvested alligators that exceeded this value. Of the counties with smaller yearly average values than Duval, two (Clay and St. Johns) border Duval and the St. Johns River. This suggests that adult alligators are relatively rare in the lower St. Johns River system and may have learned to be even more cryptic than they would be in less disturbed areas. Even more telling, nuisance alligator harvest data from FWC shows that between 2006 and 2018, average nuisance alligator total length in Duval County has steadily declined from 185 cm in 2006 to only 145 cm in 2018. Thus, juvenile and sub-adult alligators can still occupy urban areas of the lower St. Johns River system because humans are not targeting them for removal and they face virtually no competition or cannibalism from adults, while the few remaining adults appear to avoid urban areas almost entirely. The young animals are then distributing themselves at a coarse scale to minimize the negative effects of high salinity on their smaller bodies and are avoiding anthropogenic structure in favor of more natural habitat features at a finer scale. This represents a potentially significant shift in interactions between alligator size classes in urban areas relative to more natural areas.

An additional factor that likely affects alligator spatial ecology in the tributaries we surveyed is boating activity. Many of the tributaries we focused on are popular for fishing, recreational boating, and jet skiing, and crocodilians are known to avoid high-speed boat traffic^30^. Unfortunately, no boating activity data is available for the tributaries we surveyed, so we could not include it as a factor in our analyses. Future research on crocodilian spatial ecology in human-dominated areas would certainly be strengthened by explicitly incorporating boating activity into analyses.

A limitation of our study is that we only surveyed the alligator population of the lower St. Johns River system for 1 year, thus our sample size is temporally restricted. Our results would be strengthened by surveys across multiple years that would allow us to establish the consistency of the spatial ecology patterns we observed, possibly reveal other important factors that may not have been apparent over the course of our surveys, and provide insights into the potential recruitment of adults back into the population. Nevertheless, we view our study as an important first step toward understanding the effects of human activities and land development on alligators across their range, establishing a baseline that can be used for comparison with other populations.

Alligator occurrence and relative abundance across a heterogeneous habitat is multifaceted and complex, especially when considering variation between size classes and across study areas^31^. Overall, our study suggests that urban development adjacent to large river systems produces unfavorable habitat for alligators. Living in these areas has completely unknown consequences for alligator behavior, physiology, and population viability; more research is clearly needed to fully understand how these large predators may fare as urbanization continues across their range. Valuable insights could be made by studying possible differences in body condition between urban populations and those from more natural areas, along with dietary and contaminant studies. In general, large predators like alligators may actively avoid areas of human development due to habitat degradation and being targeted for removal, explaining why so few studies have been performed on large predators in urban areas.

## Materials and methods

Our work was conducted under a permit from the University of North Florida Institutional Animal Care and Use Committee (permit #18-005).

### Field methods

**Figure 1:**
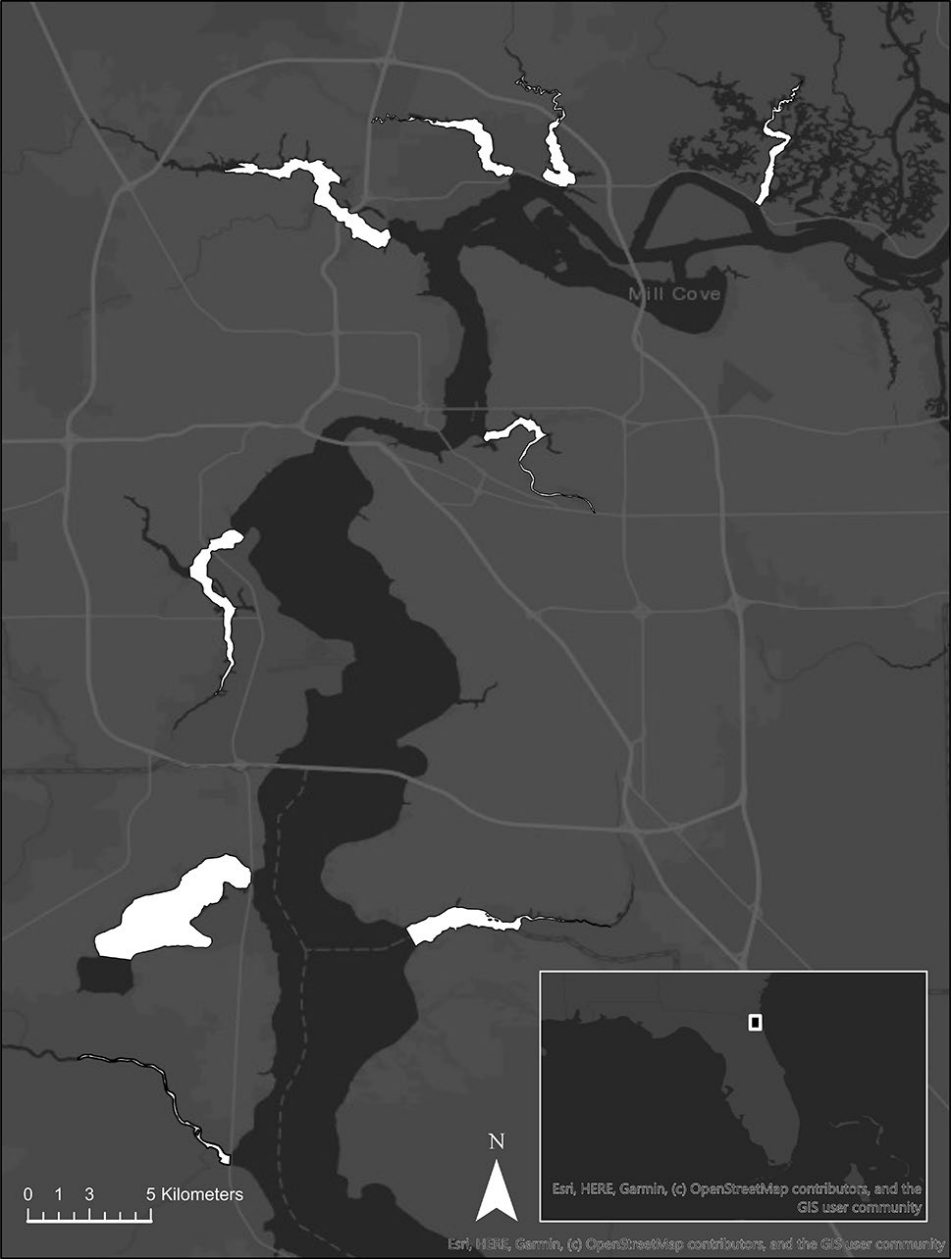
Map of the tributaries surrounding the lower St. Johns River that were surveyed as part of our study (white areas). From northeast to south: Clapboard Creek, Dunn Creek, Broward River, Trout River, Arlington River, Ortega River, Doctors Lake, Julington Creek, and Black Creek. The map was created with ArcGIS Pro 2.6 (https://arcgis.pro/).

**Figure 2:**
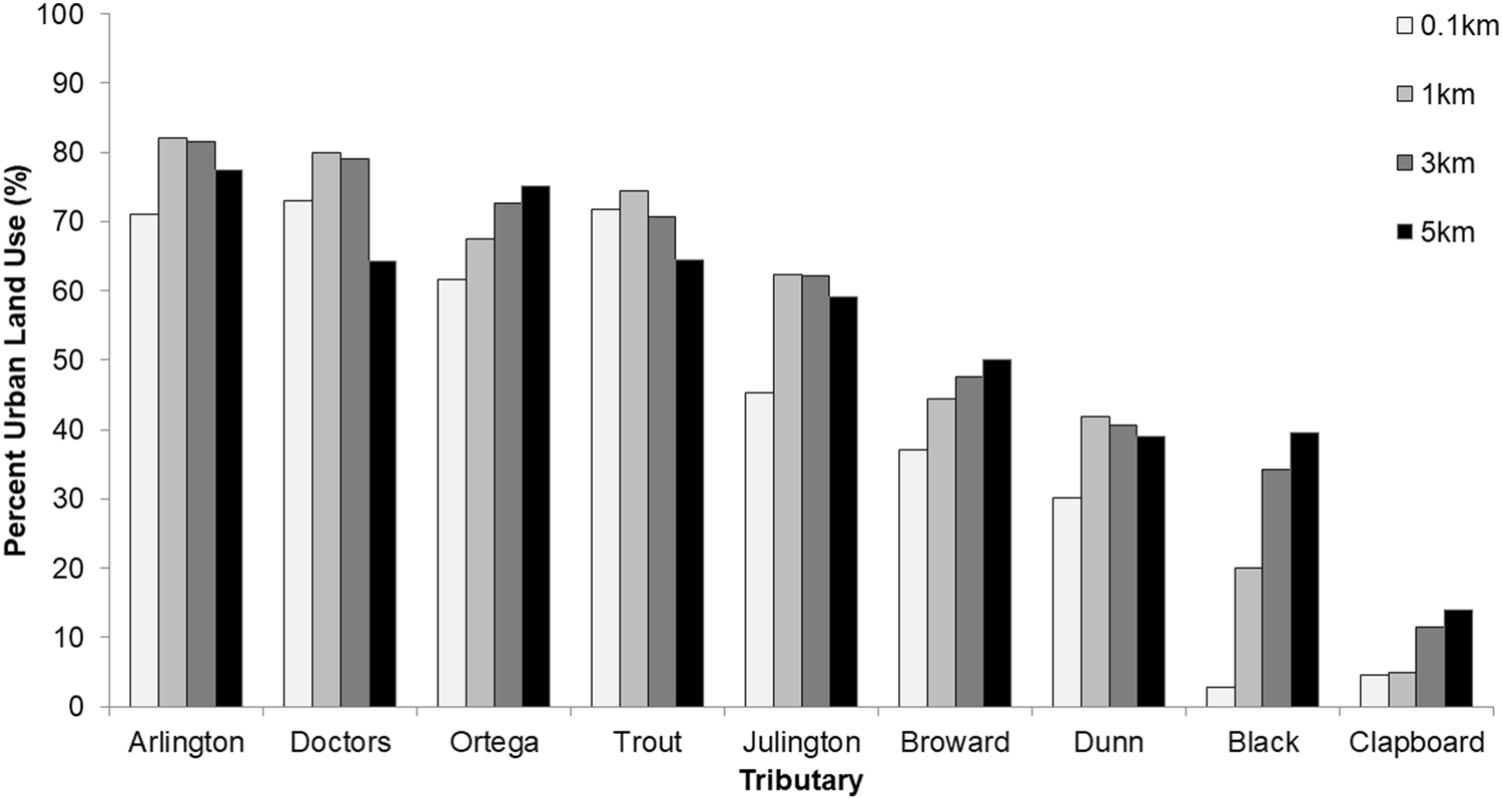
Levels of urban development (FLUCCS code 1000) surrounding the tributaries of the St. Johns River that were surveyed in this study. Land use was quantified using 0.1, 1, 3, and 5 km buffers around each tributary transect.

We performed nighttime spotlight surveys with an outboard motorboat throughout 2019 to determine relative alligator abundance, distribution, and habitat selection. This technique is an established method for estimating relative population sizes in crocodilians across heterogeneous habitat^32^. However, a limitation of spotlight surveys is the variation in detection probability caused by different environmental conditions or observers^29^. To control for these effects, we implemented a standardized survey protocol^33,34^. All surveys covered the first 8 km of nine tributaries within the lower St. Johns River system, starting at the point where each tributary meets the main channel of the river (Fig. 1). We limited our surveys to the first 8 km because some tributaries contained low bridges that blocked boat access after this point. We chose tributaries that were surrounded by different amounts of urban land cover such that our surveys spanned an urbanization gradient from approximately 5 to 80% urban land cover within 1 km of the river’s edge (Fig. 2). GIS analyses also revealed that land use patterns around the St. Johns River are dynamic, with different urban land cover proportions at 0.1, 1, 3, and 5 km from the water’s edge for each tributary (Fig. 2). To reduce temporal bias, we conducted surveys over the span of 1 year and segregated sampling periods into four distinct seasons (winter [Dec–Feb], spring [Mar–May], summer [Jun–Aug], and fall [Sep–Nov]. We surveyed each tributary one time during the middle month of each season, resulting in a total of four surveys per tributary. We surveyed the tributaries in a quasi-random fashion because the tributaries closest to the mouth of the St. Johns River are under significant tidal influence, so we timed surveys of those tributaries during periods of high tide in order to access the full survey area. We only performed surveys when rainfall was absent and wind speeds were below 16 km/h since these factors have been shown to affect alligator detection probability^24^. Quasi-random sampling over the span of a year was best suited to randomize environmental conditions that affect nighttime spotlight survey counts, such as water level, temperature, moon phase, and moon illumination^24,31,35,36^.

We began all surveys no earlier than 30 min after sunset and we maintained a constant boat speed of 10–12 km/h. At the start and end of each survey we recorded moon phase, weather conditions, visibility, ambient light, air temperature, water temperature, and salinity. We detected alligator eyeshine primarily using two 1200 lm handheld spotlights, but we also used additional handheld lights (6000 lumens) often throughout the surveys. As soon as we detected eyeshine we approached the alligator at reduced speed. We placed each individual into a size class (30–90 cm [juvenile], 90–180 cm [sub-adult], 180–270 cm [adult], 270–360 cm [large adult], + 360 cm [largest adult]) by estimating the distance between the eyes and the tip of the snout^37,38^. If an alligator submerged before size estimation could take place, we recorded its length as unknown or simply larger or smaller than 180 cm. At each sighting we recorded global positioning system location using the on-deck boat navigation unit. We measured environmental characteristics at each sighting using a YSI meter (Pro2030; YSI; Yellow Springs, Ohio, USA), a thermometer, and a sky quality meter (SQM; Unihedron; Grimsby, Ontario, Canada).

We recorded information about habitat characteristics for each sighting following previous studies^13,26^. We first visually characterized habitat in a 10 m radius circle centered on the alligator sighting location (“used habitat”). We recorded the proportion of open water, emergent vegetation, floating vegetation, anthropogenic structure, and dry ground within the circle, as well as the alligator’s distance from shore, vegetation, and anthropogenic structure. We then visually classified the same habitat characteristics in a 20 × 100 m plot centered on the alligator sighting location and stretching along the shoreline (“available habitat”). If an alligator sighting occurred entirely in open water, then we shifted the available habitat plot to the closest shoreline. For each used habitat circle and available habitat plot, we classified the respective shorelines as natural, hardened, or mixed, depending on if the shore was totally vegetated, subject to anthropogenic armoring, or a mixture of the two types, respectively. We also estimated the proportion of shoreline found within these areas that were covered in naturally growing vegetation rather than anthropogenically altered lawns.

### Land use classification

We used ArcGIS Pro (ESRI; Redlands, CA, USA) for all spatial data manipulation and visualization. We acquired land use and cover data from the St. Johns River Water Management District (SJRWMD) via the Florida Geographic Data Library. For all analyses we used data from the most recent SJRWMD dataset, which was from 2014.

We split a 100 k definition polygon of the St. Johns River to create smaller units representing each tributary transect. The resulting features consisted of the main portion of each tributary surveyed where lower order streams that were not surveyed were deleted. Because the extent to which alligators respond to land use changes was not known a priori, we buffered the transect polygon feature for each tributary to 0.1, 1, 3, and 5 km to further clip the SJRWMD land cover and use data layer. By creating four buffers for each of the nine tributaries, we generated a total of 36 land cover and use layers.

We classified land use types through the Florida Land Use and Cover Classification System (FLUCCS), as cited in SJRWMD metadata documentation. This hierarchical coding scheme contains four levels, of which we used the highest level (level 1) designation. This particular level classifies land use into nine distinct categories. These categories included urban and built-up; agriculture; upland nonforested; upland forests; water; wetlands; barren land; transportation, communication, and utilities; and special classification. For the purposes of this study, we only included defined terrestrial land use types in statistical analyses. These land use types were urban and built-up (e.g., residential, industrial, and recreational areas), agriculture (e.g., cropland, pastures, aquaculture), upland nonforested (e.g., shrub and brushland), upland forests (e.g., coniferous forests, hardwood forests, tree plantations), wetlands (e.g., freshwater/saltwater marshes, mangrove swamps, wet prairies), barren land (e.g., beaches other than swimming beaches, borrow areas, spoil areas), and transportation, communication and utilities (e.g., highways, electrical power facilities, wastewater treatment facilities). We calculated the proportions of each land use type using each respective land use shape area divided by total shape area.

### Statistical analyses

To determine if environmental conditions and/or land use characteristics affect broad scale alligator distribution, we performed multiple analyses using SPSS (IBM; Armonk, NY, USA). We included all alligator sightings in our analyses, but we did not apply population estimate correction equations to the alligator counts because they tend to underestimate population numbers in crocodilians^39^. Sighting data used in statistical analyses therefore represent relative alligator abundance, not estimates of true alligator population size. We first checked normality for each variable using Kolmogorov–Smirnov and Shapiro–Wilk tests to determine if parametric or nonparametric tests were appropriate. Normality varied greatly across the suite of variables; therefore, Spearman’s rho and Pearson’s correlation coefficient were used when appropriate. We then performed simple linear regression to determine if there were any direct relationships between relative alligator abundance and individual variables. We performed these tests for alligator counts in each tributary by season and for the average number of sightings per tributary across seasons. We also averaged environmental variables for each tributary by season and for the average value per tributary across seasons. We tested for the effect of land use at all four buffer sizes for each tributary, including all relevant terrestrial land use types.

We then performed multiple linear regression analyses in a stepwise manner. This modeling system excluded variables found to be highly correlated with other variables (multicollinear) and retained variables that significantly contributed to the model (P ≤ 0.05). We then performed these tests on modified datasets that did not contain the two most saline tributaries to further validate preliminary findings. When more than one significant model was produced for a given data set, we calculated AIC_c_ values to rank models while penalizing model complexity and accounting for our small sample sizes.

To evaluate habitat selection, we compared percent shoreline vegetation and the proportions of habitat characteristics found in the 10 m radius circle to those found in the remaining areas of each respective 20 × 100 m plot using the Wilcoxon signed rank test. When comparisons could be made between two normally distributed groups of data, we used a paired sample t test instead. While comparing used to available habitat data was the basis of the tests, the amount of data per analysis differed between analysis groups. The first group was composed of all habitat selection data across time and space. This “global” dataset was the most robust in terms of sample size but may have been biased by double counting individuals across time. The second group was divided by season, so analyses were performed on all data collected within a season across space. This group removed the bias of double counting individuals but may be affected by variation in the number of sightings per season and tributary.

## Data Availability

Raw data will be uploaded to Dryad Digital Repository upon acceptance of the manuscript for publication.
